# Hepatoprotective Effects of a Functional Formula of Three Chinese Medicinal Herbs: Experimental Evidence and Network Pharmacology-Based Identification of Mechanism of Action and Potential Bioactive Components

**DOI:** 10.3390/molecules23020352

**Published:** 2018-02-07

**Authors:** Sha Li, Ning Wang, Ming Hong, Hor-Yue Tan, Guofeng Pan, Yibin Feng

**Affiliations:** 1 School of Chinese Medicine, The University of Hong Kong, Hong Kong, China; u3003781@connect.hku.hk (S.L.); ckwang@hku.hk (N.W.); hong1986@connect.hku.hk (M.H.); hyhtan@hku.hk (H.-Y.T.); pan.gf1218@163.com (G.P.); 2 Shenzhen Institute of Research and Innovation, The University of Hong Kong, Shenzhen 518057, China; 3 Beijing Shijitang Hospital, Capital Medical University, Beijing 100069, China

**Keywords:** *Coriolus versicolor*, *Salvia miltiorrhiza*, *Schisandra chinensis*, function herbal formula, liver disease, network pharmacology

## Abstract

Various Chinese herbal medicines (CHMs) have shown beneficial liver protection effects. Jian-Gan-Bao (JGB), a functional herbal formula, consists of three famous CHMs, including *Coriolus versicolor*, *Salvia miltiorrhiza* and *Schisandra chinensis*, which has been used as a folk medicine for several chronic liver diseases. In the present study, we aim systemically to evaluate the effects of JGB on acute and chronic alcoholic liver diseases (ALD) as well as non-alcoholic fatty liver disease (NAFLD) in mouse models, and identify its potential bioactive components and mechanism of action. JGB showed preventive effects for acute and chronic ALD as well as NAFLD, while post-treatment of JGB showed no significant effect, suggesting the nature of JGB as a health supplement rather than a drug. Furthermore, a compound-target network was constructed to identify the potential bioactive compounds and pathways that regulate its hepatoprotective effects. There are 40 bioactive compounds and 15 related targets that have been identified via this network pharmacology study. Among them are miltirone, neocryptotanshinone II and deoxyshikonin, with desirable pharmaceutical properties. Pathways relating to inflammation, fatty acid oxidation, tumor necrosis factor (TNF) production and cell proliferation were predicted as bioactive compounds and potential underlying mechanisms, which should be the focus of study in this field in the future.

## 1. Introduction

Liver diseases, affecting more than 10% of the world population, remains one of the most serious public health concerns worldwide [1]. Among the various forms of liver disease, alcoholic liver disease (ALD) and non-alcoholic fatty liver disease (NAFLD) are the two most common types with high prevalence around the world [2]. ALD, resulting from chronic excessive alcohol consumption, is one of the most important causes of liver-related death, accounting for an estimated 3.8% of global mortality [3]. NAFLD, a condition caused by deposited fat in the liver generally due to a person being obese or overweight, is the most common liver disorder in developed countries. Both ALD and NAFLD may progress from simple steatosis to hepatitis, fibrosis and cirrhosis [4]. Although considerable progress has been made in understanding these pathologies, there has been little or no development of new effective therapies for these diseases [3,4,5]. Therefore, there is an urgent need for an effective and safe treatment for the prevention and treatment of ALD and NAFLD. In particular, increasingly evidence indicates that dietary health supplements, such as herbs, fruits and other natural products, play a beneficial role in preventing hepatic disorder or diseases in a major way through anti-oxidation and anti-inflammation [1].

Many herbs in Chinese herbal medicines (CHMs) are homologous with food, and have various bioactivities, such as antioxidant, anti-inflammatory, anticancer and hepatoprotective effects. Jian-Gan-Bao (JGB), a herbal combination of three famous CHMs, *Coriolus versicolor* (Yunzhi in Chinese), *Salvia miltiorrhiza* (Danshen) and *Schisandra chinensis* (Wuweizi), has been used as a complementary and alternative treatment for liver diseases [6]. Yunzhi, Danshen, and Wuweizi are three widely used herbs in CHMs. Polysaccharides are the main active components identified in Yunzhi, and possess potent anti-viral, antineoplastic, and immunomodulatory activity in vitro [7,8,9,10]. Yunzhi has been used as adjuvant therapy in cancer patients to boost human immune responses [11,12]. The hepatoprotective effects of Danshen against various pathogenic factors have been revealed in extensive studies [13,14,15,16]. Wuweizi also protects against liver diseases [17,18,19,20]. For example, biphenyl dimethyl-dicarboxylate, a compound isolated from Wuweizi, has been used for the treatment of hepatitis [21]. JGB is a generic name of the combined formula of Yunzhi, Danshen and Wuweizi. The term Jian-Gan-Bao in Chinese means liver-function improver. This combination has been commonly used as a herbal supplement to improve liver function. Therefore, the formulation consisting of these three herbs is considered to be beneficial for people with liver disorder or disease. 

Although JGB has been used in folk medicine for protection against and treatment of several chronic liver diseases, scientific evidence on its therapeutic effect is still not available. This investigation aims to systemically evaluate the effects of JGB on acute and chronic ALD as well as NAFLD in mouse models, and to explore the mechanism underlying the liver-protective effects of this formula. To further understand the action of mechanism and potential active components of JGB, we applied network pharmacology approaches in this study. Network pharmacology is an approach based on systems biology, poly-pharmacology and molecular networks, which has been extensively applied to analyze relationships between drugs and diseases in recent decade [22,23]. In particular, it has attracted considerable attention among Chinese medicine researchers for its ability to predict and illustrate interactive relationships between numerous components and targets of CHMs [24,25]. Network-based pharmacological analysis is a desirable approach for investigating the mechanisms of action for herbs and formulae and their potential bioactive components at molecular and systematic levels. In addition, it is a good tool of in silico prediction of the potential active components and action mechanisms of herbal medicines, which renders more effective subsequent exploration with experimental approaches. Network-based pharmacological analysis is a desirable approach for investigating the mechanisms of action for herbs and formulae and their potential bioactive components at molecular and systematic levels [25,26].

## 2. Results

### 2.1. The Preventive and Curative Effect of Jian-Gan-Bao (JGB) on Acute Alcoholic Liver Disease (ALD)

**Figure 1:**
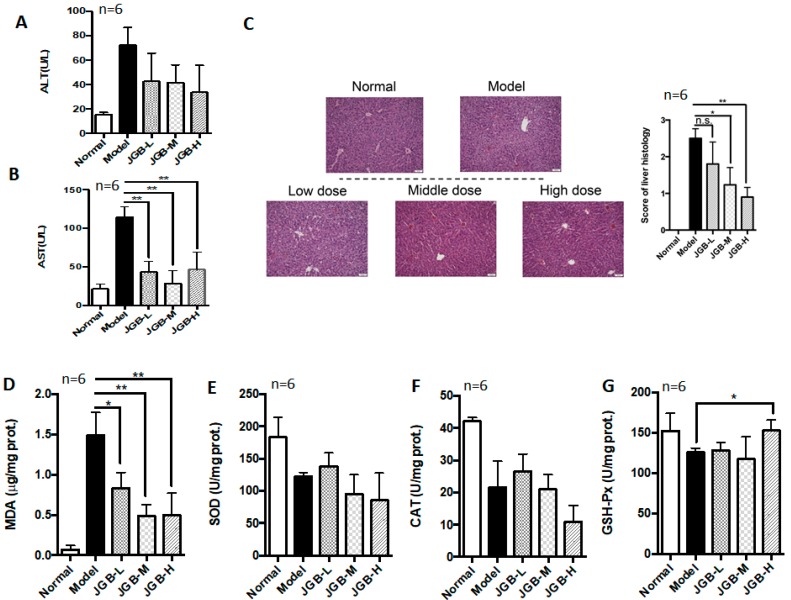
The preventive effect of Jian-Gan-Bao (JGB) on acute alcoholic liver disease (ALD). (**A**) Serum alanine aminotransferase (ALT) levels of mice from different groups; (**B**) Serum aspartate aminotransferase (AST) levels of mice from different groups; (**C**) Hematoxylin and eosin (H&E) staining images and scoring of liver histology of mice from different groups; (**D**) Malondialdehyde (MDA) levels in liver of mice from different groups; (**E**) Superoxide dismutase (SOD) levels in liver of mice from different groups; (**F**) Catalase (CAT) levels in liver of mice from different groups; (**G**) Glutathione peroxidase (GSH-Px) levels in liver of mice from different groups. (JGB-L: JGB-low dose group, JGB-M: JGB-middle group, JGB-H: JGB-high group). Three biological replicates were performed for each study. * *p* < 0.05, ** *p* < 0.01, when compared with model group.

**Figure 2:**
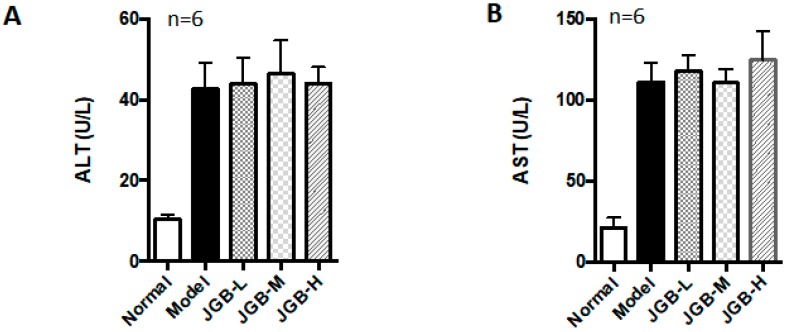

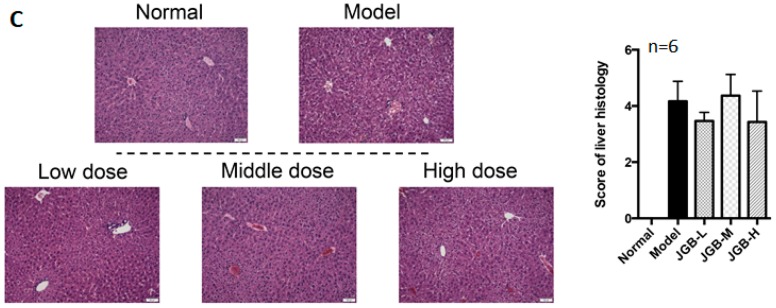
The curative effect of JGB on acute ALD. (**A**) Serum ALT levels of mice from different groups; (**B**) Serum AST levels of mice from different groups; (**C**) H&E staining images and scoring of liver histology of mice from different groups. (JGB-L: JGB-low dose group, JGB-M: JGB-middle group, JGB-H: JGB-high group). Three biological replicates were performed for each study.

The preventive and curative potential of JGB on acute ALD was studied. In the preventive treatment course, JGB exhibited protective effects against alcohol-induced liver damage in mice, as evidenced by reduced aspartate aminotransferase (AST) and alanine aminotransferase (ALT) activities as well as histological scores (Figure 1A–C). JGB could significantly reduce the lipid peroxidation end product malondialdehyde (MDA) (Figure 1D). However, we did not observe any significant activation of the internal anti-oxidative system, including superoxide dismutase (SOD), catalases (CAT) and glutathione peroxidase (GSH-Px) (Figure 1E–G). Treatment of JGB could reduce the recovery time of mice from being drunk, but it was not statistically significant (result not shown). In addition, in the curative treatment course, JGB showed non-significant therapeutic effect on liver damage (Figure 2A–C).

### 2.2. Effect of JGB on Chronic Plus Binge ALD

**Figure 3:**
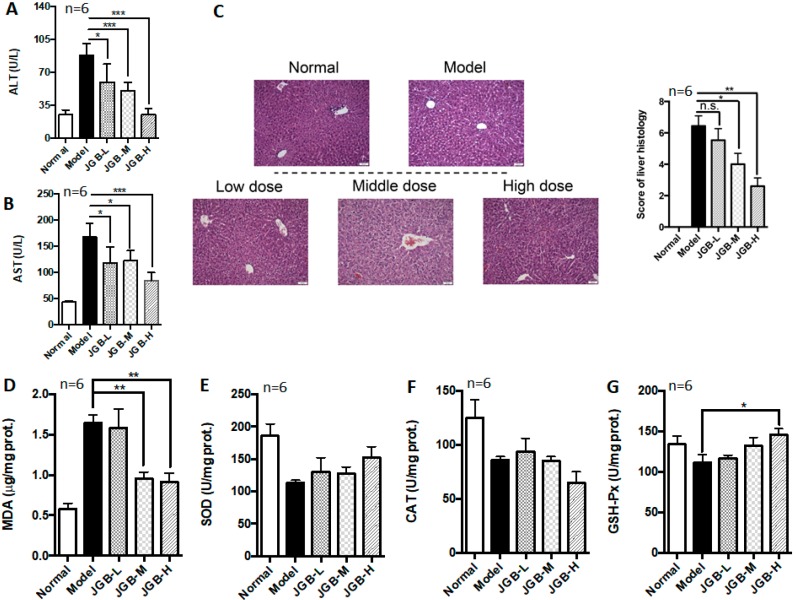
The effect of JGB on chronic ALD. (**A**) ALT levels of mice from different groups; (**B**) AST levels of mice from different groups; (**C**) H & E staining images and scoring of liver histology; (**D**) MDA levels in liver of mice from different groups; (**E**) SOD levels in liver of mice from different groups; (**F**) CAT levels in liver of mice from different groups; (**G**) GSH-Px levels in liver of mice from different groups. (JGB-L: JGB-low dose group, JGB-M: JGB-middle group, JGB-H: JGB-high group). Three biological replicates were performed for each study. * *p* < 0.05, ** *p* < 0.01, *** *p* < 0.001 when compared with model group.

After successfully establishing the chronic plus binge ALD model, the therapeutic potential of JGB on chronic ALD was studied. We observed that JGB consistently relieves chronic plus binge ALD, with reduced ALT and AST activities as well as lower histological scores in treated mice (Figure 3A–C). The microsteatosis and inflammatory cells’ infiltration was significantly relieved by JGB treatment (Figure 3C). Additionally, JGB could reduce MDA level in the liver (Figure 3D). Similar to the observation in the acute model, we found that JGB had no potent effect on the activities of SOD and CAT (Figure 3E–F). However, the activity of GSH-Px was significantly increased by high-dose treatment of JGB (Figure 3G). Overall, similar to the observation in the acute model, JGB had no potent effect on the internal anti-oxidative system of the liver.

### 2.3. The Hepato-Protective Effect of JGB on Non-Alcoholic Fatty Liver Disease (NAFLD) Combined with CCl_4_ Fibrosis

**Figure 4:**
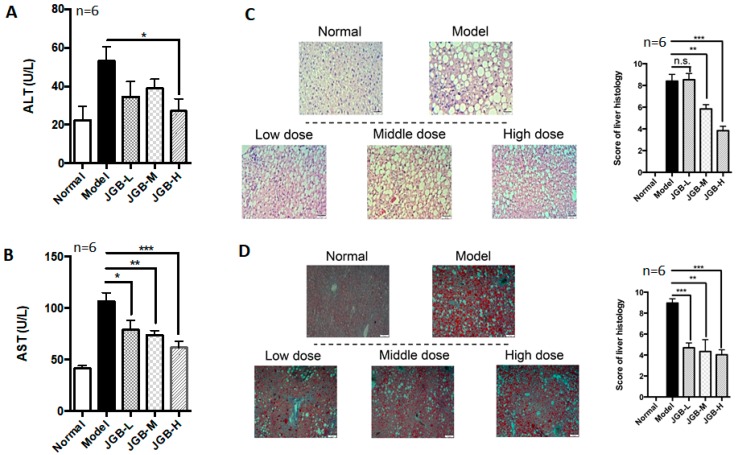

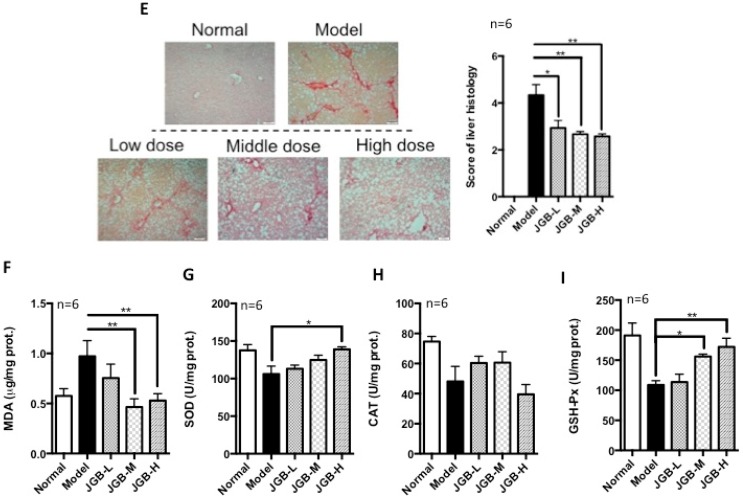
The effect of JGB on non-alcoholic fatty liver disease combined with CCl_4_ fibrosis. (**A**) Serum ALT levels of mice from different groups; (**B**) Serum AST levels of mice from different groups; (**C**) H&E staining images and scoring of liver histology of mice from different groups; (**D**) Oil-red O staining images and scoring of liver histology of mice from different groups; (**E**) Picrosirius-red staining images and scoring of liver histology of mice from different groups; (**F**) MDA levels in liver of mice from different groups; (**G**) SOD levels of mice from different groups; (**H**) CAT levels of mice from different groups; (**I**) GSH-Px levels of mice from different groups. (JGB-L: JGB-low dose group, JGB-M: JGB-middle group, JGB-H: JGB-high group). Three biological replicates were performed for each study. * *p* < 0.05, ** *p* < 0.01, *** *p* < 0.001 when compared with model group.

The therapeutic potential of JGB on NAFLD combined with CCl_4_ fibrosis was studied. The results showed that the level of ALT was significantly reduced by treatment of JGB, although the dose manner was not significant (Figure 4A). Furthermore, JGB could dose-dependently reduce AST levels in NAFLD mice (Figure 4B). Therefore, JGB possessed preventive action on the progression of fatty liver disease. The pathological features and histological scores of H&E staining indicated that liver inflammation was relieved by JGB treatment (Figure 4C). To study hepatic lipid deposition, oil-red staining was conducted. Scoring revealed results consistent with the serum test, indicating the potential of JGB in relieving fatty liver (Figure 4D). In particular, the macrovesicular steatosis as shown in the model group was obviously improved in the JGB group. As shown in Figure 4C,D, the lipidosis and adipose hollow space was reduced obviously by JGB treatment (Figure 4C,D). In mice receiving JGB administration, liver lobules and liver sinusoid were obvious, and few fat vacuoles were observed in hepatic cells. In addition, to assess if JGB could inhibit fibrosis, we conducted picrosirius-red (PSR) staining. The red stain indicated collagen deposition, which is the clinical marker of fibrosis. Reduced scores were found in JGB-treated groups, indicating that herbal medicine treatment could reduce fibrosis (Figure 4E). Hepatic MDA level was dose-dependently reduced in mice treated with JGB (Figure 4F). Treatment of JGB had minimal effects on hepatic SOD and CAT activities (Figure 4G,H). However, it could particularly induce GSH-Px (Figure 4I), indicating the involvement of anti-oxidative GSH-Px in its protective effect.

### 2.4. A Network Pharmacology Approach to Predict Potential Active Compounds and Action Mechanisms

**Figure 5:**
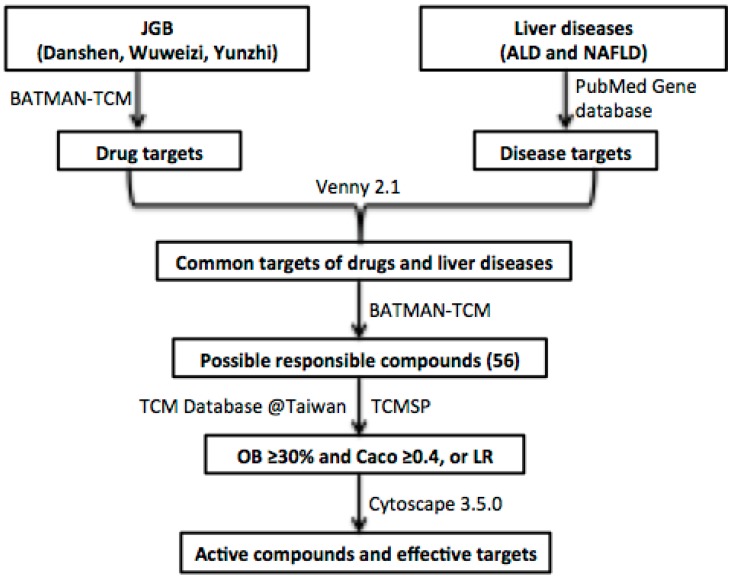
The workflow of the network pharmacological study of JGB.

**Figure 6:**
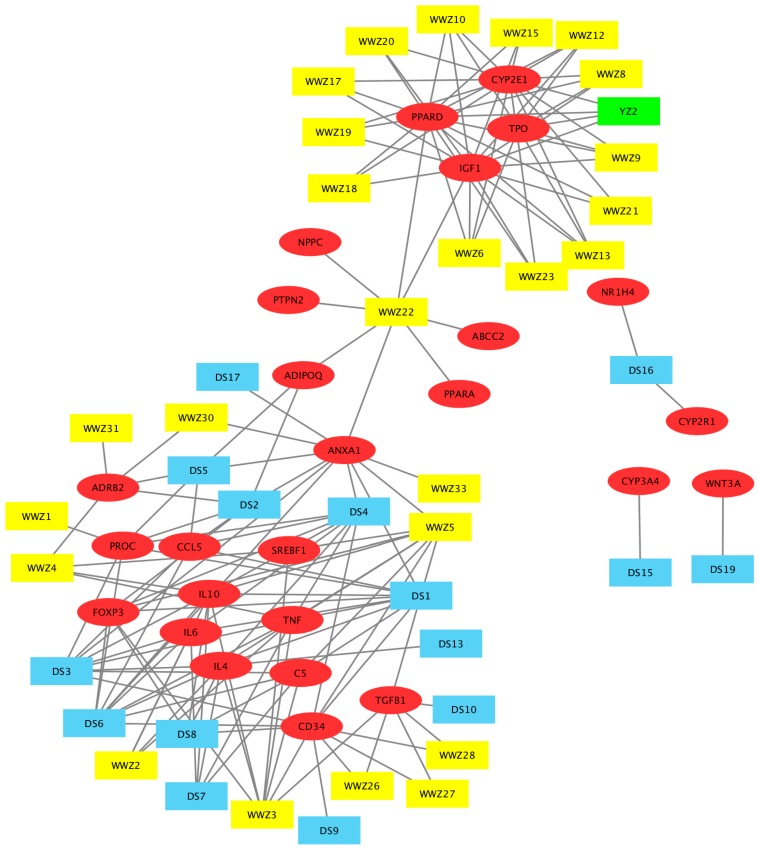
Compound-target network of JGB that is associated with ALD and NAFLD. The red ellipses represent liver disease target genes (26); the blue rectangles (14) are candidate compounds from Danshen; the yellow rectangles (25) are candidate compounds from Wuweizi; the green rectangle (1) is the candidate compound from Yunzhi; the grey lines represent the compound-target interaction. This network comprises 66 nodes (26 target genes and 40 candidate compounds). The compound codes are defined in Table 1.

**Figure 7:**
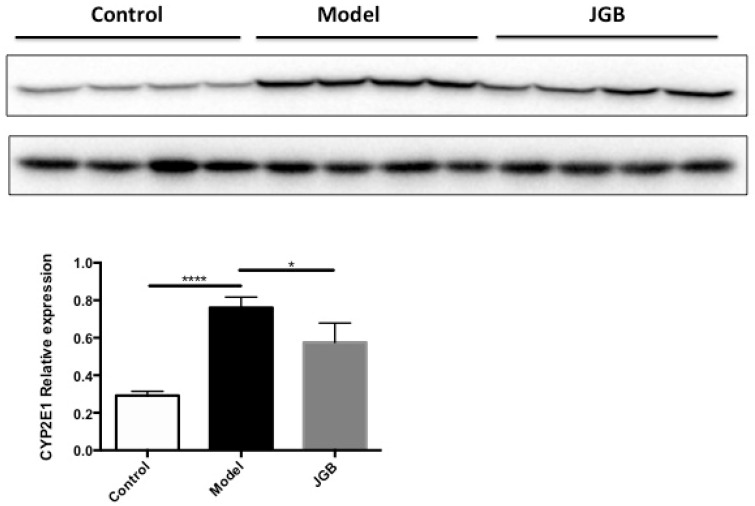
The hepatic protein level of CYP2E1 induced by ethanol was significantly decreased by JGB treatment. * *p* < 0.05, **** *p* < 0.0001 when compared with model group.

To further explore which components of JGB may be responsible for the hepatoprotective effects, a network pharmacology study was performed. The workflow of the network pharmacology analysis of JGB is shown in Figure 5. Firstly, known compound targets of Danshen (769), Wuweizi (930), and Yunzhi (319) were obtained from BATMAN-TCM (Supplemental Table S1). A total of 124 genes related to liver diseases were obtained from PubMed database (Supplemental Table S1). Then, 19 common targets in Danshen and liver diseases, 26 common targets in Wuweizi and liver diseases, 5 common targets in Yunzhi and liver diseases were recognized by VENNY 2.1 (Supplemental Figure S2). Afterwards, 56 responsible compounds in Danshen (19), Wuweizi (35), and Yunzhi (2) for these 30 targets related to liver diseases were identified based on BATMAN-TCM. Then, pharmaceutical properties analysis was performed according to their oral bioavailability (OB) and Caco-2 permeability. As suggested by the traditional Chinese medicine systems pharmacology (TCMSP) database and Hong et al. (2017), the thresholds used as active compounds screening criteria were OB ≥ 30% and Caco ≥ 0.4. For several compounds that are not included in TCMSP database but are found in TCM Database@Taiwan (http://tcm.cmu.edu. tw/), Lipinski’s Rule (LR) was used for active compound identification according to the following criteria: molecular weight (MW) ≤ 500, chemical composition with not more than ten hydrogen bond acceptors (Hacc ≤ 10), lesser than five hydrogen bond donors (Hdon ≤ 5), and octanol-water partition coefficient lesser than five (LogP ≤ 5) [22,27]. Compounds that did not satisfy at least two of the above requirements were excluded. Then 16 compounds were excluded and the remaining 40 compounds including several compounds whose phytochemical information was not available in these databases were included (Table 1). Lastly, network of compounds-targets was constructed by Cytoscape 3.5.0 (Figure 6). Based on this network, 15 potential targets were selected and their roles in liver diseases are summarized in Table 2. Furthermore, as revealed by the network pharmacological study, CYP2E1 is a potential target of JGB on liver diseases. By detecting the hepatic protein level of CYP2E1 in the ALD model, we found that the increased CYP2E1 induced by ethanol was significantly decreased by JGB treatment, consistent with our network pharmacological analysis (Figure 7).

## 3. Discussions and Future Perspectives

In this study, JGB showed preventive effects against acute and chronic ALD, as well as NAFLD. However, JGB had no significant effect after onset of acute alcoholic liver injury. This suggests that short-term administration of JGB may be inadequate for a therapeutic effect. Repeated consumption as preventive measure is suggested. This also further reflects the nature of JGB as a health supplement but not a drug. In the aspect of mechanism of action, JGB potently activated GSH-Px in NAFLD, which may partially explain its protective effect. As oxidative stress has been considered as a conjoint pathological mechanism in the initiation and promotion of liver injury, JGB might exert hepato-protective effects through anti-oxidant [28]. However, in the setting of ALD, JGB did not appear to activate the internal anti-oxidative enzymes system. As lipid peroxidation was still reduced, the pathways relating to anti-oxidative stress such as the NF-κB pathway, the sirtuin-FOXO pathway, or the Nrf-2/ARE pathway may be activated by phytochemicals of JGB, which may have cross-talk with lipid metabolism [29].

Based on the study of network pharmacology, a multitude of compounds and underlying mechanisms has been predicted. Specifically, 40 bioactive compounds and 15 related targets have been identified. Among these 40 compounds, miltirone, neocryptotanshinone II and deoxyshikonin showed very desirable pharmaceutical properties. Miltirone, a central benzodiazepine receptor partial agonist from Danshen, might interact with PROC, ADIPOQ, ADRB2, FOXP3, ANXA1, and CCL5, which may deliver hepatoprotective effects through mediating inflammation and fatty acid oxidation, leading to the reduced lipid peroxidation observed in our experimental data. Another compound, neocryptotanshinone II, known as 6,12-dihydroxyabieta-5,8,11,13-tetraen-7-one, is a diterpenoid isolated from Danshen [30]. Structurally, it is very similar to neocryptotanshinone, which possesses anti-inflammatory properties [31,32]. They might also have the similar physicochemical, biological and pharmacological properties. In this network pharmacology study, it is shown to interact with many vital targets including PROC, IL10, IL6, TNF, IL4, Foxp3, CD34, ANXA1, and CCL5. 5,8-Dihydroxy-2-(4-methyl-3-pentenyl)naphthoquinone, commonly known as Deoxyshikonin, was initially identified from *Lithosperraum erythrorhizon* Sieb. et Zucc and was also found to present in Wuweizi. Deoxyshikonin is considered to be a promising drug candidate for treatment of lymphatic diseases and wound healing [33], but its application in liver diseases is still not available. Interestingly, it can also interact with PROC. PROC, a type of anticoagulant protein, is usually used as a marker in determining the severity of the liver disease [34]. It is the only common gene targeted by Danshen, Wuweizi, and Yunzhi. We have also found that the hepatoprotective effect of JGB was better than single herb (data not shown), suggested the collective action of these compounds on PROC. It was reported that PROC was elevated in patients having fatty liver diseases due to increased hepatic insulin resistance. These phytochemicals, miltirone, neocryptotanshinone ii and deoxyshikonin might regulate lipid metabolism through mediating PROC [34], which should be validated by subsequent experimental approaches. Moreover, among these 15 associated proteins, ADIPOQ, ADRB2, FOXP3, IL10, PPARD, and PROC could negatively regulate inflammatory response; CD34, ADIPOQ, FOXP3, and IL10 are negative regulators of tumor necrosis factor; ANXA1, IGF1, IL6, PPARD, and TNF are associated with cell proliferation. Particularly, targets of ADIPOQ, CYP2E1, IL6, SREBF1, and TNF are related to NAFLD. The interaction of herbal compounds on these targets and predicted pathways deserves to be studied in detail in further study. Furthermore, as revealed by the network pharmacological study, CYP2E1 is a potential target of JGB on liver diseases. Particularly, CYP2E1 plays a vital role in ALD. CYP2E1 could be induced by alcohol, which is an effective generator of reactive oxygen species such as hydrogen peroxide and superoxide anion radical, and produces powerful oxidants such as the hydroxyl radical, resulting in hepatic oxidative stress [3]. Thus, we speculated that CYP2E1 might be the primary targets of JGB in ALD. By detecting the hepatic protein level of CYP2E1 in the ALD model, we found that the increased CYP2E1 induced by ethanol was significantly decreased by JGB treatment, consistent with our network pharmacological analysis. In addition, for the future translational study of JGB, individual factors, such as genetic predisposition and co-morbidities, should be carefully considered. Generally, inter-individual differences of patients result in different responses to pharmacological treatment [35,36]. Individual factors, such as genetic predisposition and co-morbidities, play an important role in acute and chronic liver diseases [35]. Thus, the impact of individual factors—which include physiological factors such as age, gender and concomitant diseases, genetic factors, and environmental factors including co-administered medications, diet, and smoking behavior—on the therapeutic effect of JGB in liver diseases deserves to be noted and highlighted [37].

## 4. Materials and Methods

### 4.1. Reagents

Ethanol (99.9%) and carbon tetrachloride (CCl_4_, 99.9%) were purchased from Thermo Scientific (Waltham, MA, USA). Liquid ethanol diet and control liquid dextrose diet were bought from Bio-Serv (Flemington, NJ, USA). Choline-deficiency amino acid-defined diet (CDAA) was obtained from Research Diets (New Brunswick, NJ, USA). Kits for alanine transaminase (ALT, catalogue #2930), aspartate transaminase (AST, catalogue #2920) were bought from Medex Supply (Passaic, NJ, USA). Kits for malondialdehyde (MDA, catalogue #A003-1), superoxide dismutase (SOD, catalogue #A001-1), glutathione peroxidase (GSH-Px, catalogue #A005), and catalase (CAT, catalogue #A007-1) were bought from Nanjing Jiancheng (Nanjing, China). Direct red 80, picric acid and oil-red O were purchased from Sigma-Aldrich (St. Louis, MO, USA). The JGB aqueous extract was obtained from Vitagreen (Hong Kong, China), which was produced by mixing the three herbs together and extracting by indicated solvents with standard operation in good manufacturing practice (GMP) manufacturers.

### 4.2. Animal Treatment

Male C57BL/6J mice 5 weeks weighting 20 ± 2 g were obtained from Laboratory Animal Unit of The University of Hong Kong. We had 6 mice in each group, and ketamine (100 mg/kg)/xylazine (10 mg/kg xylazine) were used to anaesthetize the animals before sacrifice. All animal experimental protocols were approved by the Committee on the Use of Live Animals in Teaching and Research (CULATR) of The University of Hong Kong, Hong Kong (document number: 3646-15).

To investigate the preventive effect of JGB on acute ALD, mice were given JGB extract at the dose of 280, 840 and 2520 mg/kg via gavage for 9 days. Control and model groups of mice were treated with an equal volume of water. Then 6 h after the last treatment, mice received ethanol (6 g/kg via gavage). The normal group of mice received isocaloric/isovolumetric maltose-dextrin solution at the same volume. Recovery time from drunkeness was recorded. Then 9 h after ethanol treatment, mice were sacrificed, and samples including blood and liver were collected immediately.

The curative effect of JGB on acute ALD was studied as follows. Mice were treated with ethanol (6 g/kg via gavage) while the control group received isocaloric/isovolumetric maltose-dextrin solution at the same volume. Mice were given JGB extract via gavage 2 h later. Then, after 9 h, mice were sacrificed, and samples including blood and liver were collected immediately.

To induce chronic-plus-binge alcoholic liver injury, we used the protocol as described by Bertola et al. [38]. Briefly, mice were initially fed with control liquid dextrose diet for 5 days for acclimation. Afterwards, the mice received a liquid ethanol diet for 11 days. On the last day, mice were given a single dose of ethanol (5 g/kg) via gavage. Normal mice received the control liquid dextrose diet for 11 days. For the herbal medicine treatment, mice were given JGB extract via gavage daily throughout the experiment. Similarly, 9 h after the final ethanol treatment, mice were sacrificed to collect blood and liver samples immediately.

For the establishment of the NAFLD model, mice were fed with a control chow or CDAA diet for 6 weeks. A low dose of CCl_4_ (0.4 μL/kg b.w., twice/week) was used to promote fibrosis. As the CDAA diet combined with CCl_4_ injection mimics the pathological spectrum of chronic liver disease well, we applied this model thereafter to study the effects of JGB on the progress from liver steatosis to fibrosis. For the herbal medicine treatment, mice were given JGB extract via gavage daily throughout the experiment. At the endpoint, mice were sacrificed, and samples including blood and liver were collected immediately.

### 4.3. Biochemical Assays

Blood samples were centrifuged at 3000 rpm for 10 min and the serum samples obtained were stored at −20 °C for further analysis. The contents of ALT and AST in the serum were detected by commercial kits. The procedures were conducted as described by the manufacturers’ instructions. Briefly, ALT and AST working reagents were prepared according to instructions and then 100 μL serum samples were added to 1mL working reagent. Units per liter (U/L) of ALT/AST activity is the amount of enzyme which oxidizes one μmol/L of NADH per minute. Namely, the average absorbance per minute was determined, multiply by factor −1746 for results in U/L. The activities of CAT, GSH-Px, and SOD as well as the production of MDA of liver tissues were determined by kits bought from Nanjing Jiancheng. The decomposing reaction of H_2_O_2_ by CAT could be terminated by ammonium molybdate, and resting H_2_O_2_ reacts with ammonium molybdate to produce faint yellow complex compound with maximum absorption at 405 nm. The determination of GSH-Px is based on the oxidation of glutathione (GSH) to oxidized glutathione (GSSG) catalyzed by GSH-Px, which is then coupled to the recycling of GSSG back to GSH utilizing glutathione reductase (GR) and NADPH. Since GSH-Px is the rate-limiting factor of the coupled reactions, the decrease in NADPH absorbance measured at 340 nm during the oxidation of NADPH to NADP^+^ is indicative of GSH-Px activity. The SOD assay Kit-WST conveniently allows SOD assaying by utilizing WST-1 (2-(4-Iodophenyl)-3-(4-nitrophenyl)-5-(2,4-disulfophenyl)-2*H*-tetrazolium that generates a water-soluble formazan dye upon reduction with a superoxide anion. SOD inhibits the rate of the reduction with O_2_, which is linearly related to the xanthine oxidase activity. Therefore, the 50% inhibition activity of SOD (IC50) can be determined by a colorimetric method. The measuring principle of MDA is that the red product generated by the condensation reaction between MDA and TBA has maximum absorption at 532 nm. 

### 4.4. Liver Histology

For the H&E staining study, the liver tissues were fixed in 10% buffered formaldehyde, and then cut into 5 μm thick paraffin sections by a Leica RM 2016 rotary microtome (Leica Instruments Ltd., Shanghai, China). The tissues were stained with hematoxylin and eosin staining (H&E staining). The liver damage was assessed by the following criteria: 0, no obvious injury; 1–3, mild injury with inflammatory infiltration; 4–6, intermediate injury with microvesicular steatosis or intermediate necrosis; 7–9, severe injury with structural disorder of hepatic lobules, or ‎macrovesicular steatosis; and 10, hepatic structure destruction. For hepatic lipid accumulation analysis, oil-red O staining was performed. Cryostat sections of liver tissues were cut into 10 μm thickness and then stained with oil red O. Hepatic steatosis scoring on the stained sections was made by three individual examiners with the criteria as described in previous paper [23,39]: 0, no droplet; 1–3, rare to few small droplet; 4–6, moderate small droplet; 7–9, large droplet; and 10, whole stain. Additionally, picrosirius-red staining in liver tissue was conducted to evaluate liver fibrosis. Briefly, 5 μm thick paraffin sections were stained with picrosirius red. Scoring on the stained sections was made according to the following criteria: 0, no obvious fibrosis signs; 1–3, no portal area fibrosis; 4–6, fibrosis appears in the portal area; 7–9, a destroyed lobule structure fibrosis without cirrhosis; 10, fibrosis and cirrhosis [23].

### 4.5. Western Blot

Total protein was extracted from liver tissue of mice via RIPA lysis buffer supplemented with phosphatase inhibitor. The supernatant was transferred and equal amounts of protein were separated on sodium dodecyl sulfate polyacrylamide gel electrophoresis (SDS-PAGE) gel and blotted onto polyvinylidene difluoride membranes. The membrane was then incubated with CYP2E1 (ab28146) and GAPDH primary antibodies at 4 °C overnight followed by incubation with appropriate horseradish peroxidase (HRP) labelled secondary antibodies. The blots were subjected to chemiluminescence analysis (Bio-rad, Hercules, CA, USA).

### 4.6. Network Pharmacology Construction and Analysis

The constituent compounds and related targeted genes of these three herbs (Danshen, Wuweizi, Yunzhi) were identified by BATMAN-TCM (Bioinformatics analysis tool for molecular mechanism of traditional Chinese medicine, http://bionet.ncpsb.org/batman-tcm/). Liver disease targets including ALD and NAFLD were collected from PubMed Database (https://www.ncbi.nlm.nih.gov/pubmed/). The common targets were recognized by VENNY 2.1 (http://bioinfogp.cnb.csic.es/tools/venny/). Then, the network of compounds-targets was constructed by Cytoscape 3.5.0. The pharmaceutical properties’ analysis was performed according to Lipinski’s rule (LR) and the absorption, distribution, metabolism, and excretion (ADME) system. 

### 4.7. Statistical Analysis

All experiments were performed in triplicate. All the data obtained is presented as the mean ± SD, and analyzed by one-way analysis of variance (ANOVA) with SPSS. Value of *p* < 0.05 was considered to be statistically significant. 

## 5. Conclusions

This study found that JGB showed preventive effects against acute and chronic ALD as well as NAFLD, while post-treatment with JGB had no significant effect, suggesting the nature of JGB as a health supplement rather than a drug. In our NAFLD model, JGB can specifically activate GSH-Px, which may be partially involved in the protective effect. However, the internal anti-oxidative system including SOD and CAT in the context of ALD are not activated, indicating that JGB does not protect the liver from alcohol-induced injury by alleviating oxidative stress. It is expected that JGB may regulate the lipid metabolism of an injured liver to prevent hepatic damage, fatty liver and fibrogenesis, which needs to be further studied. Based on the construction of network pharmacology, 40 bioactive compounds and 15 related targets have been identified as possible effective compounds and potential targets. Among them, miltirone, neocryptotanshinone II and deoxyshikonin are compounds with good pharmaceutical properties and that act with vital targets of pathways relating to inflammation, fatty acid oxidation, TNF production and cell proliferation, suggesting that they should be the focus of future study on this formula. The results provide scientific support for developing this formula, Jian-Gan-Bao, as a functional supplement for the prevention of alcoholic liver injury and NAFLD.

## Figures and Tables

**Table 1:** The 56 compounds from Danshen, Wuweizi, and Yunzhi and their corresponding molecular properties, OB and Caco.

Phytochemical	MW	AlogP	Hdon	Hacc	OB (%)	Caco-2	Herb No.
Tanshiquinone B	280.318	3.769	1	3			DS1
Miltirone	282.41	4.73	0	2	38.76	1.23	DS2
Miltirone I	312.36	3.353	1	4			DS3
Dehydromiltirone	280.39	4.29	0	2	24.57	1.14	DS5
Neocryptotanshinone II	270.35	3.61	1	3	39.46	0.76	DS6
Neotanshinone C	252.265	3.061	1	3			DS4
Ferruginol	286.452	6.004	1	1			DS7
Isotenulin	306.354	1.804	0	5			DS8
Dihydrokaranone *	218.37	4.17	0	1	28.86	1.39	DS9 *
Salvinone							DS10
Salviol *	302.50	4.74	2	2	24.31	0.95	DS11 *
Danshensu *	198.19	0.71	4	5	36.91	‒0.27	DS12 *
1-Hydroxytaxinine A	492.55	1.453	2	9			DS13
Cryptoxanthin *	552.96	10.76	1	1	25.16	1.84	DS14 *
Heteratisine	391.50	0.454	2	6			DS15
Gamma-Sitosterol	414.70	8.084	1	1			DS16
Isocucurbitacin D	516.67	1.647	4	7			DS17
Ursolicacid *	456.78	6.47	2	3	17.7	0.56	DS18 *
Samaderin A	330.33	0.054	1	6			DS19
Vitamin B2 *	376.41	0.23	5	10	6.79	−1.22	YZ1 *
Carotene							YZ2
Deoxyshikonin	272.32	3.50	2	4	73.85	0.74	WWZ1
Nonylphenol							WWZ2
(*E*)-9-Isopropyl-6-Methyl-5,9-Decadiene-2-One	208.34	4.065	0	1			WWZ3
Epiguaipyridine	215.37	3.95	0	1	36.98	1.66	WWZ4
Nootkatone	218.37	3.61	0	1	33.04	1.36	WWZ5
1-Methyl-4-Methylethenylcyclohexene							WWZ6
Gamma-Selinene *	204.39	4.95	0	0	22.58	1.84	WWZ7 *
Beta-Chamigrene	204.39	4.71	0	0	31.99	1.82	WWZ8
Clovene	204.39	4.09	0	0	46.49	1.80	WWZ9
Thujopsene	204.39	4.08	0	0	53.81	1.85	WWZ10
Beta-Bisabolene *	204.39	5.33	0	0	29.59	1.88	WWZ11 *
Beta-Pinene	136.26	2.93	0	0	44.77	1.85	WWZ12
Beta-Sesquiphellandrene	218.42	5.39	0	0	30.58	1.82	WWZ13
Myrcene *	136.26	3.69	0	0	24.96	1.84	WWZ14 *
Alpha-Limonene	136.26	3.5	0	0	39.84	1.83	WWZ15
Epsilon-Cadinene *	204.39	4.85	0	0	16.41	1.82	WWZ16 *
D-Limonene							WWZ17
Camphene	136.26	2.93	0	0	34.98	1.81	WWZ18
Isolongifolene	204.39	4.08	0	0	46.32	1.8	WWZ19
Longifolene	204.39	4.18	0	0	39.49	1.83	WWZ20
Sesquicarene							WWZ21
Clupanodonic Acid	276.46	5.50	1	2	44.01	1.22	WWZ22
1-(1,5-Dimethyl-4-Hexenyl)-4-Methyl Benzene	208.38	5.775	0	0			WWZ23
Isolychnose *	664.60	−7.593	14	20			WWZ24 *
Elemicin *	208.28	2.79	0	3	21.94	1.41	WWZ25 *
Terpinen-4-Ol	154.28	2.55	1	1	81.41	1.36	WWZ26
Delta-Terpineol	154.28	2.47	1	1	55.11	1.28	WWZ27
Linalool	170.28	1.43	1	2	49.73	0.86	WWZ28
3-Phenyldecane *	218.42	6.22	0	0	4.73	1.89	WWZ29 *
Phenyl-2-Propanone	134.19	1.46	0	1	39.66	1.32	WWZ30
Phenylpropyl Alcohol	136.21	2	1	1	36.57	1.18	WWZ31
Campherenol *	222.41	3.80	1	1	18.25	1.35	WWZ32 *
1-Phenyl-1,3 Butanedion	162.18	1.48	0	2			WWZ33
Longispinogenin *	472.83	5.31	3	3	16.07	0.35	WWZ34 *
Myricadiol *	442.80	6.21	2	2	13.58	0.78	WWZ35 *

Note: * compound does not satisfy the screening criteria.

**Table 2:** The information of major potential targets of JGB in liver diseases.

Target Gene	Target Protein	Biological Effects Associated with Liver Diseases
*CYP2E1*	Cytochrome P450 2E1	Oxidative stress and fatty acid oxidation
*PPARD*	Peroxisome proliferator-activated receptor delta	Integrator of transcription repression and nuclear receptor signaling, lipid accumulation
*IGF1*	Insulin-like growth factor 1	Activators of the AKT signaling pathway, cell death
*TPO*	Thyroid peroxidase	Primary regulator of platelet production
*ANXA1*	Annexin 1	Anti-inflammation
*ADIPOQ*	Adiponectin	Modulates glucose regulation and fatty acid oxidation
*PROC*	Protein C	Anticoagulant protein, marker in determining the severity of the liver disease
*CCL5*	Chemokine CCL5	Mediate the interaction between immune cells and hepatic stellate cells
*FOXP3*	Forkhead box P3	Regulatory T cells, immune cells regulation
*IL10*	Interleukin-10	Modulate Kupffer cells, liver inflammation and fibrosis
*IL6*	Interleukin-6	Pro-inflammation and anti-inflammation
*SREBF1*	Sterol regulatory element-binding transcription factor 1	Glucose metabolism, fatty acid and lipid production
*TNF*	Tumor necrosis factor	Activates several intracellular pathways to regulate inflammation, cell death, and proliferation
*CD34*	CD34 protein	Facilitate cell migration, dedifferentiation
*TGF-β*	Transforming growth factor-β	Inflammation, wound healing, hepatocyte damage, hepatocyte proliferation, fibrogenesis

## Supplementary Materials

The following are available online.

## Abbreviations

The following abbreviations are used in this manuscript:

ADIPOQ	Adiponectin
ALD	Alcoholic liver diseases
ALT	Alanine aminotransferase
ANXA1	Annexin 1
AST	Aspartate aminotransferase
CHMs	Chinese herbal Medicines
CAT	Catalases
CCL5	Chemokine CCL5
CD34	Cluster of differentiation 34
CYP2E1	Cytochrome P450 2E1
FOXP3	Forkhead box P3
GSH-Px	Glutathione peroxidase
IGF1	Insulin-like growth factor 1
IL10	Interleukin-10
IL6	Interleukin-6
JGB	Jian-Gan-Bao
MDA	Malondialdehyde
MW	Molecular weight
NAFLD	Non-alcoholic fatty liver disease
OB	Oral bioavailability
PPARD	Peroxisome proliferator-activated receptor delta
PROC	Protein C
SOD	Superoxide dismutase
SREBF1	Sterol regulatory element-binding transcription factor 1
TGF-β	Transforming growth factor-β
TNF	Tumor necrosis factor
TPO	Thyroid peroxidase
