# Supplementary material for: Hepatoprotective Effects of a Functional Formula of Three Chinese Medicinal Herbs: Experimental Evidence and Network Pharmacology-Based Identification of Mechanism of Action and Potential Bioactive Components
Source: Molecules. 2018 Feb 7;23(2):352. doi: 10.3390/molecules23020352 (PMC6017312; doi:10.3390/molecules23020352)
Supplement: Supplementary file 1 [file molecules-23-00352-s001.zip › Supplemental table 1-Liver diseases targets.docx]

| Symbol | Aliases |
| --- | --- |
| Apoe | AI255918, Apo-E |
| Tnf | DIF, TNF-a, TNF-alpha, TNFSF2, TNFalphaa, Tnfsf1a, Tnlg1f, Tnf |
| Tlr4 | Lps, Ly87, Ran/M1, Rasl2-8 |
| Lep | ob, obese |
| Tgfb1 | TGF-beta1, TGFbeta1, Tgfb, Tgfb-1 |
| Il6 | Il-6 |
| Myd88 |  |
| Akt1 | Akt, LTR-akt, PKB, PKB/Akt, PKBalpha, Rac |
| Shh | 9530036O11Rik, Dsh, Hhg1, Hx, Hxl3, M100081 |
| Pparg | Nr1c3, PPAR-gamma, PPAR-gamma2, PPARgamma, PPARgamma2 |
| Pecam1 | C85791, Cd31, PECAM-1, Pecam |
| Casp3 | A830040C14Rik, AC-3, CASP-3, CC3, CPP-32, CPP32, Caspase-3, Lice, SCA-1, Yama, mldy |
| Nfe2l2 | Nrf2 |
| Bdnf |  |
| Ptgs2 | COX2, Cox-2, PES-2, PGHS-2, PHS II, PHS-2, Pghs2, TIS10, gripghs |
| Il17a | Ctla-8, Ctla8, IL-17, IL-17A, Il17 |
| Igf1 | C730016P09Rik, Igf-1, Igf-I |
| Tnfrsf1a | CD120a, FPF, TNF-R, TNF-R-I, TNF-R1, TNF-R55, TNF-alphaR1, TNFAR, TNFR60, TNFRI, TNFRp55, TNFalpha-R1, Tnfr-2, Tnfr1, p55, p55-R |
| Myc | AU0167572, Niard, Nird, bHLHe39, Myc |
| Ppara | 4933429D07Rik, AW742785, Nr1c1, PPAR-alpha, PPARalpha, Ppar |
| Il1b | IL-1beta, Il-1b |
| Ccl2 | AI323594, HC11, JE, MCAF, MCP-1, MCP1, SMC-CF, Scya2, Sigje |
| Sirt1 | AA673258, SIR2L1, Sir2, Sir2a, Sir2alpha |
| Ifnar1 | CD118, Ifar, Ifnar, Ifrc, Infar |
| Gsk3b | 7330414F15Rik, 8430431H08Rik, C86142, GSK-3, GSK-3beta, GSK3 |
| Hmox1 | D8Wsu38e, HO-1, HO1, Hemox, Hmox, Hsp32 |
| Trpv1 | OTRPC1, TRPV1alpha, TRPV1beta, VR-1, Vr1 |
| Nlrp3 | AGTAVPRL, AII/AVP, Cias1, FCAS, FCU, MWS, Mmig1, NALP3, Pypaf1 |
| Rela | p65 |
| Ccr2 | Cc-ckr-2a, Ccr2b, Ckr2, Ckr2a, Ckr2b, Cmkbr2, mJe-r, Ccr2 |
| Adipoq | 30kDa, APN, Acdc, Acrp30, Ad, GBP28, adipo, apM1 |
| Jun | AP-1c, c-jun, Jun |
| Il18 | Igif, Il-18 |
| Il1r1 | CD121a, CD121b, IL-1R1, IL-iR, Il1r-1 |
| Insr | 4932439J01Rik, CD220, D630014A15Rik, IR, IR-A, IR-B |
| Serpine1 | PAI-1, PAI1, Planh1 |
| Cd36 | FAT, GPIV, Scarb3 |
| Stat5a | AA959963, STAT5 |
| Prkaa1 | AI194361, AI450832, AL024255, AMPKalpha1, C130083N04Rik |
| Prkcd | AI385711, D14Ertd420e, PKC[d], PKCdelta, Pkcd |
| Npc1 | A430089E03Rik, C85354, D18Ertd139e, D18Ertd723e, lcsd, nmf164, spm |
| Scarb1 | AI120173, CD36, Cd36l1, Chohd1, Cla-1, Cla1, D5Ertd460e, Hdlq1, Hlb398, SR-B1, SR-BI, SRBI, Srb1, mSR-BI |
| Nr1h3 | AU018371, LXR, RLD1, Unr1 |
| Ikbkb | AI132552, IKK-2, IKK-beta, IKK2, IKK[b], IKKbeta |
| Tlr7 |  |
| Ccr5 | AM4-7, CD195, Cmkbr5 |
| Ppard | NUC-1, NUC1, Nr1c2, PPAR-beta, PPAR-delta, PPAR[b], PPARdelta, Pparb, Pparb/d |
| Nanog | 2410002E02Rik, ENK, ecat4 |
| Cdc42 | AI747189, AU018915 |
| Nr1h4 | AI957360, Fxr, HRR1, RIP14, Rxrip14 |
| Apoa1 | Alp-1, Apoa-1, Brp-14, Ltw-1, Lvtw-1, Sep-1, Sep-2, Sep2, apo-AI, apoA-I |
| Mir155 | Mirn155, mir-155, mmu-mir-155 |
| Lcn2 | 24p3, AW212229, NRL, Sip24 |
| Gh | Gh1b1, Gh |
| Vcam1 | CD106, Vcam-1 |
| Hnf4a | HNF-4, Hnf4lpha, MODY1, Nr2a1, TCF-14, Tcf14, Hnf4a |
| Adam17 | CD156b, Tace |
| Lrp1 | A2mr, AI316852, CD91, Lrp, b2b1554Clo |
| Srebf1 | ADD1, SREBP-1a, SREBP1, SREBP1c, bHLHd1 |
| Epas1 | HIF-2alpha, HIF2A, HLF, HRF, MOP2, bHLHe73 |
| Irf3 | C920001K05Rik, IRF-3 |
| Tgfa | wa-1, wa1 |
| Scd1 | AA589638, AI265570, Scd, Scd-1, ab |
| Id1 | AI323524, D2Wsu140e, Idb1, bHLHb24 |
| Nr1h2 | AI194859, LXR, LXRB, LXRBSV, LXRbeta, NER1, OR-1, RIP15, UR, Unr, Unr2 |
| Mc4r | Mc4-r, Pkcp |
| Fgf21 |  |
| Sele | CD62E, E-selectin, ELAM-1, Elam, LECAM2 |
| Nt5e | 2210401F01Rik, 5'-NT, AI447961, CD73, NT, Nt5, eNT |
| Cyp2e1 | Cyp2e |
| Alox15 | 12-LO, 12/15-LO, 15-LOX, Alox12l, L-12LO |
| Il1rn | F630041P17Rik, IL-1ra |
| Hsd11b1 |  |
| Nr1i2 | PXR, PXR.1, PXR.2, PXR1, SXR, mPXR |
| Timp3 | Timp-3 |
| Otc | AI265390, Sf, spf |
| Yy1 | AW488674, NF-E1 |
| Abcb4 | Mdr2, Pgy-2, Pgy2, mdr-2 |
| Vldlr | AA408956, AI451093, AW047288 |
| Tmem173 | 2610307O08Rik, ERIS, MPYS, Mita, STING |
| Retn | ADSF, Fizz3, Rstn, Xcp4 |
| Bmp6 | D13Wsu115e, Vgr1 |
| Plin2 | AA407157, ADPH, Adfp, Adrp |
| Fabp1 | Fabpl, L-FABP |
| Meis1 | C530044H18Rik, Evi8 |
| Casp2 | CASP-2, ICH-1, NEDD-2, Nedd2 |
| Tph1 | Tph |
| Nr5a2 | AU020803, D1Ertd308e, Ftf, LRH-1, UF2-H3B |
| S100a8 | 60B8Ag, AI323541, B8Ag, CFAg, CP-10, Caga, MRP8, p8 |
| Mir34a | Mirn34a, mir-34a, mmu-mir-34a |
| Cyr61 | AI325051, CCN1, Igfbp10 |
| Gpnmb | DC-HIL, Dchil, ipd |
| Fasn | A630082H08Rik, FAS |
| Sirt6 | 2810449N18Rik, AI043036, Sir2l6 |
| Rbp4 | Rbp-4 |
| Cxcl9 | BB139920, CMK, Mig, MuMIG, Scyb9, crg-10 |
| Adipor1 | 2810031L11Rik, ACDCR1, CGI-45, Paqr1 |
| Rgn | AI265316, GNL, SMP30 |
| Gas6 | Gas-6 |
| Cyp2a5 | CYPIIA5, Coh, Cyp15a2 |
| Adamts1 | ADAM-TS1, ADAMTS, ADAMTS-1, C3-C5, METH-1, METH1 |
| Mc3r | MC3-R |
| Fech | AI894116, Fcl, fch |
| Adh1 | ADH-AA, AI194826, Adh-1, Adh-1-t, Adh-1e, Adh-1t, Adh-3e-e, Adh1-t, Adh1tl, Adh3-e, Adh1 |
| Mir122 | Mir122a, Mirn122a, mir-122, mmu-mir-122 |
| Abcg5 | cmp, sterolin-1, trac |
| Pemt | PEAMT, PEMT2, PLMT, Pempt, Pempt2 |
| Abcg8 | 1300003C16Rik, AI114946, sterolin-2 |
| Aoc3 | SSAO, VAP1 |
| Scp2 | AA409774, AA409893, C76618, C79031, NSL-TP, SCP-2, SCP-X, SCPX, ns-LTP |
| Tnfrsf10b | DR5, KILLER, Ly98, MK, TRAILR2, TRICK2A, TRICK2B, TRICKB |
| Klf6 | AI448727, BCD1, C86813, CPBP, Copeb, FM2, FM6, Ierepo1, Ierepo3, R75280, Zf9 |
| Adipor2 | 1110001I14Rik, ADCR2, AI115388, AW554121, D6Ucla1e, Paqr2 |
| Abcb11 | ABC16, Bsep, Lith1, PFIC2, PGY4, SPGP |
| Cpb2 | 1110032P04Rik, 4930405E17Rik, AI255929, CPR, Cpu, TAFI |
| Cmklr1 | ChemR23, DEZ, Gpcr27, mcmklr1 |
| Srd5a1 | 0610031P22Rik, 4930435F02Rik, S5AR 1, Srd5a-1 |
| Cyp2b10 | Cyp2b, Cyp2b20, p16 |
| Ctf1 | CT-1 |
| Npc1l1 | 9130221N23Rik, Gm243 |
| Cdc25a | D9Ertd393e |
| Mir26a-1 | Mirn26a, Mirn26a-1, miR-26a, mir-26a-1 |
| Sult1e1 | EST, ST1E1, Ste |
| Dgat2 | 0610010B06Rik, ARAT, DGAT-2 |
| Map3k11 | 2610017K16Rik, Mlk3, PTK1, SPRK |
| Lats2 | 4932411G09Rik, AV277261, AW228608 |
| Elovl6 | C77826, FAE, LCE |
| Prtn3 | PR3, mPR3 |
| Serpina1a | Aat-2, Aat2, Dom1, PI1, Spi1-1, Spi1-3 |
| Gstm1 | Gstb-1, Gstb1 |
| Igf2bp2 | C330012H03Rik, IMP-2, Imp2, Neilsen |
| Srsf3 | AL024116, Sfrs3, X16 |
| Tpcn2 | D830047E22Rik, Gm35086 |
| Loxl2 | 1110004B06Rik, 4930526G11Rik, 9430067E15Rik |
| Litaf | 3222402J11Rik, C85531, N4WBP3, TBX1 |
| Lrpprc | 3110001K13Rik, C76645, Gp130, Lrp130, Lsfc |
| Sirt7 |  |
| Blvra | 0610006A11Rik, 2500001N03Rik, Blvr |
| Pdk2 |  |
| Pnpla3 | Adpn |
| Hdac8 | 2610007D20Rik |
| Sidt2 | B930096O19, BC023957, CGI-40 |
| Atp10a | Atp10c, pfatp |
| Cic | 1200010B10Rik, mKIAA0306 |
| Gtf2h1 | 62kDa, AW743425, AW822074, BTF2 p62, C77871, p62 |
| Dpp9 | 6430584G11Rik, A330078I11, DPP IX, DPRP2 |
| Maf1 | 1110068E11Rik, AU042856 |
| Fgl1 | Mfire1 |
| Hsd17b13 | AI047820, PAN1B-like, Pan1b |
| Tnfrsf23 | SOB, TNFRSFH23, Tnfrh1, Tnfrsf1al1, mDcTRAILR1, mSOB |
| Dpp8 | 2310004I03Rik, 4932434F09Rik, AI666706, DPP VIII |
| Mir291b | Mirn291b, mir-291b, mmu-mir-291b |
| NEWENTRY |  |
