# Supplementary figures and images for: Hepatoprotective Effects of a Functional Formula of Three Chinese Medicinal Herbs: Experimental Evidence and Network Pharmacology-Based Identification of Mechanism of Action and Potential Bioactive Components
Source: Molecules. 2018 Feb 7;23(2):352. doi: 10.3390/molecules23020352 (PMC6017312; doi:10.3390/molecules23020352)

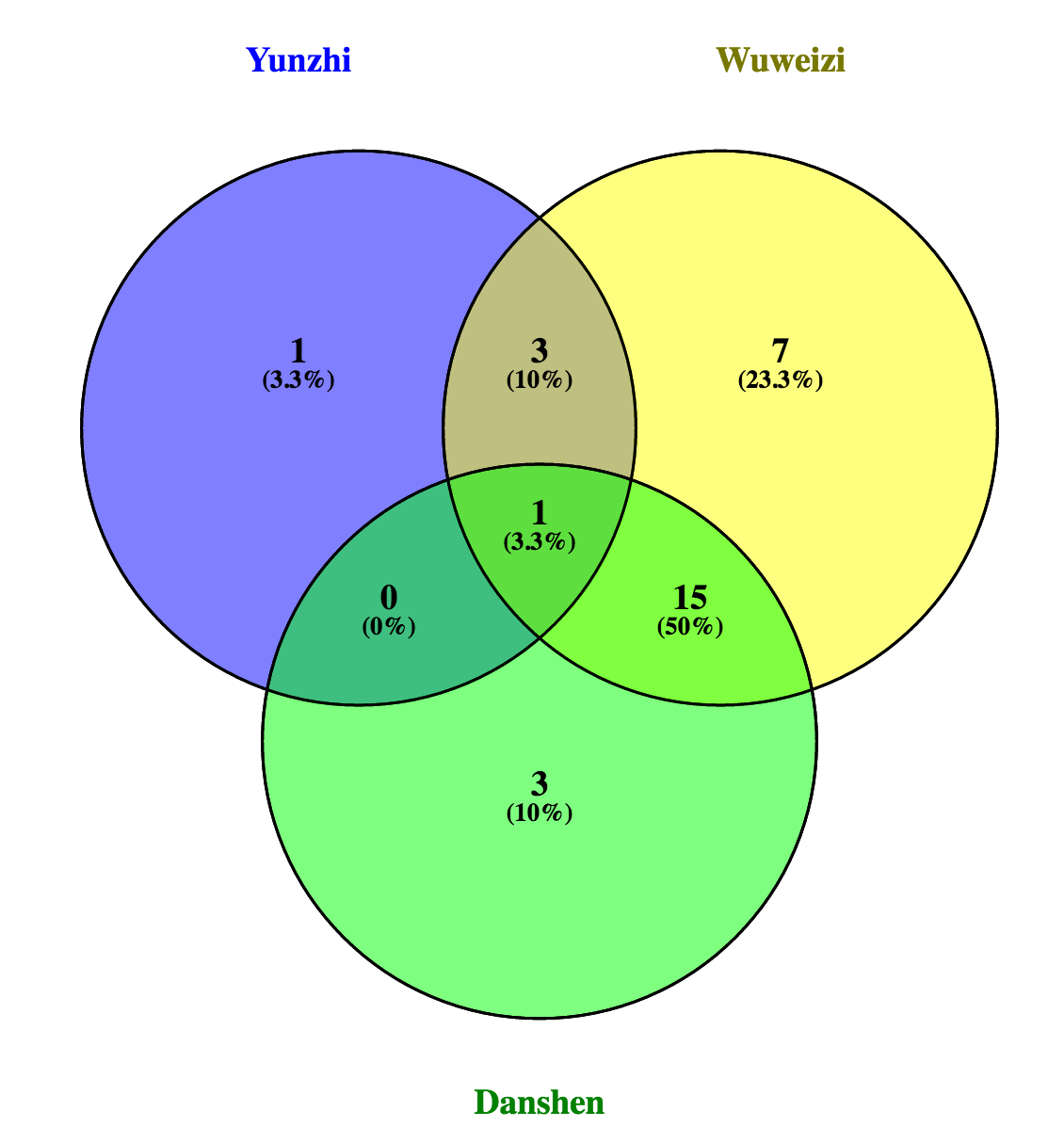

Supplement: Supplementary file 1 [file molecules-23-00352-s001.zip › Supplemetal Figure 2.png]
